# Whole body radiotherapy: A TBI-guideline

**DOI:** 10.4103/0971-6203.25664

**Published:** 2006

**Authors:** Ulrich Quast

**Affiliations:** University Hospital, Essen, Germany

**Keywords:** TBI, dosimetry, guidelines

## Abstract

Total Body Irradiation (TBI) is one main component in the interdisciplinary treatment of widely disseminated malignancies predominantly of haematopoietic diseases. Combined with intensive chemotherapy, TBI enables myeloablative high dose therapy and immuno-ablative conditioning treatment prior to subsequent transplantation of haematopoietic stem cells: bone marrow stem cells or peripheral blood progenitor stem cells. Jointly prepared by DEGRO and DGMP, the German Society of Radio-Oncology, and the German Association of Medical Physicists, this DEGRO/DGMP-Leitlinie Ganzkoerper-Strahlenbehandlung - DEGRO/DGMP Guideline Whole Body Radiotherapy, summarises the concepts, principles, facts and common methods of Total Body Irradiation and poses a set of recommendations for reliable and successful application of high dose large-field radiotherapy as essential part of this interdisciplinary, multi-modality treatment concept. The guideline is geared towards radio-oncologists, medical physicists, haematooncolo-gists, and all contributing to Whole Body Radiotherapy. To guide centres intending to start or actualise TBI criteria are included. The relevant treatment parameters are defined and a sample of a form is given for reporting TBI to international registries.

Based on recommendations prepared by DGMP working group 2 - *Physical Aspects of Total Body Irradiation* - this guideline on *Whole Body Radiotherapy* takes into account recommendations of AAPM Task Group 29/AAPM-Report No. 17, *Total and Half Body Radiotherapy,*[1] from review articles on physical aspects of TBI;[2–11] on clinical and biological aspects of TBI;[12–23] on HSCT[24–30] and from TBI-symposia.[31–36,11] This German TBI-guideline www.DEGRO.org, and www.DGMP.de[37] were confined to issues of high dose TBI. The present guideline will not address the indications and principles of hematopoietic stem cell transplantation nor those of low dose TBI, an experimental strategy with a dose of about 2 Gy, in test for immuno-ablative conditioning, with promising results.

## 1. Concept of total body irradiation

*High Dose Therapy (HDT)* combines high dose *Total Body Irradiation (TBI)* and intensive chemotherapy with subsequent allogeneic, autologous or syngeneic transplantation of HLA-identical hematopoietic stem cells (HSCT) - bone marrow stem cells (BMT) or peripheral blood progenitor stem cells (PBPST) and supportive care under sterile conditions during the aplastic phase. The indications for TBI are listed in Table 1, the tasks of TBI in Table 2 and the complications of HDT-HSCT in Table 3.

**Table 1:** Indications for TBI

**Certain indications:** Leukaemias in adults and childhood: - Acute lymphoblastic leukaemia (ALL), - Acute myeloid leukaemia (AML), - Chronic myeloid leukaemia (CML), - Myelodysplastic syndrom (MDS). **Optional indications:** Solid tumors in childhood: - Neuroblastomas, - Ewing sarcomas, - Plasmocytomas / multiple myelomas. **In clinical test:** - Morbus Hodgkin's disease (MHD) - Non-Hodgkin's lymphomas (NHL).

**Table 2:** Tasks of TBI

**Myeloablative therapy:**
The irreversible elimination of the clonogenic malignant cells is the therapeutic task of high dose TBI in treatment of widely disseminated malignancies.
**Immunoablative conditioning treatment:**
The induction of immuno-suppression is the conditioning task of TBI in allogeneic hematopoietic stem cell transplantation to enable successful engraftment.

**Table 3:** Complications of HDT-HSCT

***Early reversible toxicity of radio-chemotherapy (partly with a high risk of lethality):***
Nausea and vomiting, mucositis (oropharyngeal, gastro-intestinal), renal malfunction, arrhythmia, haemorrhagic cystitis, bone marrow aplasia, infections, haemorrhage, interstitial pneumonitis, alveolary haemorrhage, veno occlusive disease, cardiomyopathy, alopecia, nail growth disorder, parotitis.
**Late toxicity of radio-chemotherapy:**
Endocrine and reproductive gonadal insufficiency, growth disorders (in childhood), hypothyreosis, lung fibrosis, cataract, secondary malignomas, irreversible alopecia, cardiomyopathia.
**Immunologic complications after HSCT:**
Graft rejection, acute/chronic graft-versus-host-disease (GvHD), persistent risk of infections.

*Target volume of high dose TBI* is the whole body, including the skin, as the *target cells* are widely disseminated, all manifest or occult clones of malignant cells, including those circulating and the whole cellular immune system. Organs with a high risk of recurrence (“homing phenomenon”) and primary extended (“bulky”) tumour regions like meninges, testes, or abdominal lymph nodes may require additional concurrent local radiotherapy.

*Organs at risk in HDT/HSCT* are lungs, kidney, liver, spleen, heart and eyes, endangered by the cumulative toxicity of combined treatment (sequential radio-chemotherapy and prophylactic treatment). Other factors of risk are previous diseases and treatments, the type of donor and transplant and infections (e.g, cytomegalovirus, herpes simplex virus, mycosis). As the irradiation of the whole hematopoietic system causes failure of myelopoiesis and the associated complications, the whole course of treatment has to be performed under conditions of low risk of infections, especially in allogeneic transplantation.

*Sparing of organs at risk* with an almost parallel functional architecture needs to maintain the organ function in a sufficiently large partial volume; for the lungs: by reduction of the organ dose, the total dose (by shielding) and the single dose (by fractionation), limiting the effective dose rate and avoiding interactions of radiation, chemotherapy and prophylactic treatment (choice of drug and dosage schedule). Although experimental data gives evidence for a higher toxicity of chemotherapy prior to TBI, in clinical application the sequence is assumed to play a minor role, especially in allogeneic HSCT. After fractionation, most complications known from single fraction TBI do not occur, or occur later and with a lower frequency and severity such as cataracts or interstitial pneumonitis. Nausea and vomiting can be managed anticipatively. Organs with an almost serial functional architecture are spared by avoiding any over-dosage.

## 2. Preconditions for total body irradiation

*Close interdisciplinary cooperation* of radio-oncologists, medical physicists and hematooncologists is the basis for successful TBI as part of HDT prior to HSCT. Myeloablative HDT and transplantation should therefore be performed in the same center, enabling joint consultations on the concept and course of treatment, on patient care and follow-up. The roles and responsibilities have to be clarified and documented. TBI should be performed only in large centers (awaiting a high frequency of patients - best > 20 per year) and having sufficient qualified personnel (including trained stand-ins), premises and technical support.

*Reliability:* TBI must be reliable. To guarantee TBI, an emergency concept must be prepared for staff and equipment with an alternative method available and ready to continue TBI. Reliable equipment is required for providing precise and comfortable positioning of the patient and exact delivering of the prescribed TBI dose distribution to target volume and organs at risk. Dedicated TBI treatment rooms and facilities are suitable in centers with a high TBI frequency. Suitable equipment for localisation (CT, simulator), planning and preparation of shieldings; moulages or fixation aids; suitable dosemeters; and phantoms must be available.

## Reasonability: *TBI should be simple and clear with reasonable costs and workload*

***Evaluability:*** HDT results are of interest for evaluation. Thus, all beams and dose modifications required to achieve the prescribed distribution of dose in target volume and organs at risk must be applied in each fraction, as required by radiobiological reasons. Serial application of TBI techniques, with different sets of parameters in different fractions, cannot be evaluated, or can be evaluated only with limitations. A uniform way of dose specification is crucial.

***Precision:*** Optimal TBI requires individual treatment planning based on systematic dose measurements and CT-localisation under treatment conditions, considering tissue inhomogeneities and individual body contours, careful performance of TBI with verification and control and documentation of all relevant treatment parameters.

***Dose homogeneity:*** For TBI, pairs of opposing high energy photon beams are used. While the anterior-posterior (a.-p.) separations vary little along the body, typically between 20 and 25 cm for adults, the lateral body separations show large variations with maximal values up to about 50 cm for adult patients. Thus, already the AAPM-Report No. 17 recommends an a.-p.-p.-a. -TBI-technique,[1] providing to realize dose precision and homogeneity easily. Accelerator photons of 4-6 MeV maximal energy and ^60^Co-gamma-radiation yield good opposing beam depth dose homogeneity (≤ ± 3% between both dose maxima and about ± 7% including the skin). ^60^Co-treatment units are also reliable, with a low rate of breakdown and lesser time required for maintenance and quality assurance. Dose homogeneity can also be achieved by suitably combining concurrent bilateral and a.-p.-p.-a.-TBI. For bilateral TBI alone, photons of at least 10 MeV maximum energy are required (depth dose inhomogeneities of about ± 10%; up to ± 30% including skin dose). Additional dose modifications to adjust the low dose to skin, head, neck and legs are needed too.

***Reproducibility:*** A stretched supine and prone position should be chosen. This enables CT localisation in treatment position. For long-term irradiation, remotely positioned dose modifiers are not recommended.

***Effectiveness of lung shielding:*** Individually shaped partially transmitting shields of calculated thickness reduce the lung dose as prescribed. In a.-p.-p.-a.-TBI, shielding is most effective without under-dosage of too many target cells and is very precise if shields are directly fixed to the skin. Bilateral TBI alone causes a low dose in mediastinum, ribs and arms.

***Technical requirements:*** It might be necessary to modify the treatment facility (if allowed): dose monitor range, additional flattening filter, correction for dose rate variations, or inclusion of all TBI devices into the safety circuit. Electronic verification should control all parameters determining the TBI dose. The position of shields has to be verified under treatment conditions prior to each beam. A film-developing unit should be available close by. An emergency kit (including reanimation means) is a must to be prepared for complications of lung, circulation or heart. A puls-oxymeter can supervise the patients' body functions.

## 3. Performance of total body irradiation

**Figure 1:**
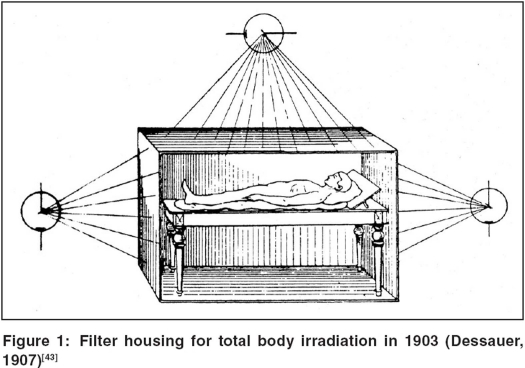
Filter housing for total body irradiation in 1903 (Dessauer, 1907)[43]

Since 100 years [Figure 1], numerous techniques have been developed worldwide to perform Total Body Irradiation utilising photon radiation. Recommendations for a special TBI technique can however not be given as the frequency of treatments is too low to reach the statistical significance for conclusions from clinical results.[23]

**Figure 2:**
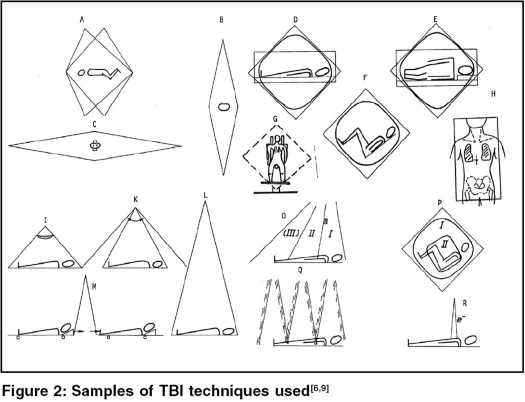
Samples of TBI techniques used[6,9]

*Reliability, evaluability, reasonability, precision, dose homogeneity, reproducibility and the effectiveness of lung shielding* - these criteria should be considered if TBI is introduced or changed. Table 4 shows typical TBI techniques that have been in use, listed according to radiation incidence, beam direction, patient position, number of opposing beams and timing schedule. No evidence is found that simultaneous irradiation of all parts of the body with identical dose rate (requiring two or more radiation sources) is demanded [Figure 2 ABC].

**Table 4:** Techniques of total body irradiation [Figure 2]

a.-p. - p.-a.-TBI with “vertical” beams
Simultaneous a.-p. - p.-a.-TBI - in supine position - utilising two dedicated opposing beam radiotherapy treatment units with wide angle collimators; two stories needed (**B**). a.-p. - p.-a.-TBI - in prone & supine position - utilising opposing beams in a large treatment distance (≥6 m); requiring a two story treatment room below or above the accelerator (**L** or inverse to **L**). a.-p. - p.-a.-TBI - in prone & supine position - utilising opposing large beams (wide angle collimator & flattening filter needed) in a short distance (< 2m); suitable in small rooms (**I**). a.-p. - p.-a.-TBI - under patient translation in prone and supine position - utilising opposing beams in short distance (<2 m); suitable in small rooms, with diagonal coach position even in very small rooms (**M**). Multi-Strip- a.-p. - p.-a.-TBI - in prone and supine position - utilising opposing, overlapping, parallel beams in short distance (<2 m); used as emergency-TBI; suitable in small rooms. Sweeping beam- a.-p. - p.-a.-TBI - in a stretched or bended prone & supine position - utilising sweeping opposing beams at short distance (<2 m); suitable in small rooms (**K**). a.-p. - p.-a.-TBI - in prone & supine position - utilising multiple beams (3 to 4 adjacent beams from different directions) in short distance (<2 m); suitable in small rooms (**O**). a.-p. - p.-a.-TBI - in prone & supine position - utilising multiple (3 to 4) parallel opposing beams in short distance (<2 m); suitable in small rooms (**Q**).
**a.-p. - p.-a.-TBI with horizontal beams**
Simultaneous a.-p. - p.-a.-TBI - the patient lying on the side - utilising two treatment units. A dedicated, very large extended treatment room is required (**C**). a.-p. - p.-a.-TBI - the patient lying on the side - utilising opposing beams at large distance (4-6 m). A large room is required (**D**). a.-p. - p.-a.-TBI - in a “standing” position - utilising opposing beams at large distance (4-6 m). A large room is required (**G**).
**Bilateral TBI with horizontal beams**
Simultaneous bilateral TBI - in a stretched supine position - utilising opposing beams from 2 treatment units in very large distance. An extended treatment room is required (**C**). Bilateral TBI - in a stretched supine position - utilising opposing beams in large distance (4-6 m). An extended treatment room is required (**D**). Bilateral TBI - in a “sitting” position - utilising opposing beams in medium distance (3-4 m). A large treatment room is required (**F, P**).
**Concurrent bilateral and a.-p. - p.-a.-TBI (in each fraction)**
Bilateral and a.-p. - p.-a.-TBI - in a stretched supine position or the patient lying on the side - utilising two pairs of opposing horizontal beams in large distance (4-6 m; extended treatment room is required), applied in each fraction (**D, E**). Bilateral and a.-p. - p.-a.-TBI - in a “sitting” or supine position - utilising horizontal and vertical opposing beams (or multiple beams) in large distance (4-6 m; extended treatment room is required), applied in each fraction (**F, O**).

***Half body irradiation (HBI):*** For some palliative indications such as the generalised prostate carcinoma with osseous metastases, where myelopoiesis has to be kept, a sequential upper and lower half body irradiation with opposing beams can be applied taking the diaphragm as border and keeping a pause of 6 weeks to allow for bone marrow regeneration.

***Total nodal / lymphoid irradiation (TNI / TLI):*** For some indications, such as aplastic anemia, concurrent a.-p.-p.-a.-irradiation of the whole supra- and infradiaphragmal lymph node system is indicated, with the mean position of the diaphragm as border.

***Local radiotherapy:*** Concurrent local irradiation may be indicated to extended primary tumour sites, e.g, to the testes (e.g, 6 Gy with 3 fractions in 3 days), or to the central nervous system (CNS); due to the high cumulative toxicity of HDT, only the brain should be irradiated, not the spine. Pre-irradiation of the spine can be a counterindication for TBI; its tolerance could be exceeded.

## 4. Irradiation treatment planning

*TBI is required in HDT*, especially in the treatment of acute leukemias [Table 1].

***Optimization:*** TBI treatment planning has to guarantee a sufficiently high and homogeneous dose in the target volume under optimal sparing of the lungs, the organ most at risk. Reported TBI-treatment parameters, even those from clinical trials, can often not be compared or transferred. They are defined differently and inconsistently. Very different values are in use for the dose, dose rate and fractionation,[9] even today.

***Prescription, recording and reporting TBI:*** It is very difficult to determine optimal relevant TBI parameters from clinical data, as combined HDT/HSCT is too complex and the number of comparable treatments too small to evaluate the exact contribution of radiation therapy parameters.[18,21,38,23] Therefore, no general recommendations can be given.

***Dose specification:*** In TBI, all organs at risk are also part of the target volume. Thus, optimization of treatment efficiency and the avoidance of complications are strongly coupled and require dose prescription for both, the dose to the target volume and the reduced dose to the lungs, organ most at risk. In optimization of dose prescription, the target dose should not be increased when requirement is to further reduce the lung dose.[13] The total TBI reference dose, ***D_Ref_***, should not be essentially higher than 12 Gy. Although higher doses may contribute to improved control of the disease, trials have provided with evidence that higher doses cause a rapid increase in the rate of severe complications as well.[39–42,23] In radiotherapeutic prescription, also the number of fractions and the lung dose rate have to be specified. Single fraction TBI has been left as too many complications have been observed. In fractionated TBI, the total dose is increased by 20-25% compared to single fraction TBI.

Centers starting TBI or changing over to TBI should take a dosage and fractionation concept that is approved with a large number of patients and should keep this as long as the clinical results are sufficiently good. Numerous dosage and fractionation schemes have been applied for TBI. Typical examples of schedules in use in Germany are:

- ***D_Ref_ = 10 Gy,*** ***D_Lung_ = 8 Gy*** *in 4 fractions on 4 consecutive days with single doses* ***d_Ref_= 2.5 Gy, d_Lung_= 2 Gy;*** (applied without change since 1983 for about 2,000 patients at the Essen University Hospital, largest German center with > 100 TBI patients a year).
- ***D_Ref_ = 12 Gy, D_Lung_*** = *7; 8; 8.5; 9; 9.5; 10;* or even *11 Gy* in *6 fractions on 3 consecutive days, 2 fractions a day* with a gap of 4-6 h and 16-18 h respectively. This scheme with *6 fractions of* *d_Ref_ = 2.0 Gy,* is used in most German centers, however, with quite different lung doses.

**Figure 3:**
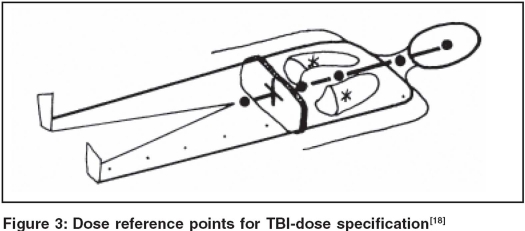
Dose reference points for TBI-dose specification[18]

*The relevant parameters of TBI:* The reference point (+) for dose specification to the target volume is defined at mid-abdomen at the height of the umbilicus according to an international consensus [Figure 3]. The lung dose is defined as the mean of the dose at the midpoints of both lungs (*), [Figure 3]. This dose value corresponds to the minimum dose to the lungs as the lung dose increases towards the borders due to scatter from surrounding tissues. The spatial distribution of dose in the target volume can be characterized by the dose-volume histogram (DVH) or by a triplet of dose values: (*D_Ref_, D_min_, D_max_*), the dose at the reference point (+) and the dose variation in the target volume. The dose variation can be derived from the longitudinal dose homogeneity (at selected reference points (·) along the body midline) and the transverse dose homogeneity (the minimum and maximum dose in beam directions within a plane through the dose specification point and containing the beam axis) [Figure 3]. The lung dose and the DVH, or the relative shielded part of the lungs (e.g, 70%), characterize the spatial distribution of dose within the lungs. The temporal distribution is characterized[18] by stating the overall irradiation time (including the first day), the number of fractions and the biologically effective lung dose rate (the lung dose per fraction divided by the lung irradiation time per fraction.[14] Table 5 shows a form for reporting TBI data to EBMT, the European Bone Marrow Transplant registry; and to IBMTR, the international registry.

**Table 5:** Reporting the relevant TBI parameters to EBMT[18]

*Patient ID:*……………..		*………………. *Clinical diagnosis:*	……………………………………….

T B I		Total body irradiation	
I	**Target volume TBI dose_______**	**Gy**	***Whole body, incl. skin Dose specification point: mid abdomen at height of umbilicus***
	HOMOGENEITY	DOSE-VOLUME-HISTOGRAM DOSE-VARIATION	*Needs 3D-planning, or*
		a) Transverse: + ___ - ___ %	*Across specification point*
		b) Longitudinal: + ___ - ___ %	*Along body mid-line*
		ORGAN DOSES	*Partial target volume*
		_________________: _________ Gy _________________: _________ Gy _________________: _________ Gy	*DD/D ≥ ±10% of TBI-dose due to shieldings or additional beams*

II	**Normal tissues LUNG DOSE _____**	**LUNGS Gy,**	***Organ most at risk Mean of total dose at centre of both lungs***
		DOSE-VOLUME-HISTOGRAM SHIELDED VOLUME ________ %	*Needs 3D-planning, or Estimated*
	**FRACTIONS ______**	**in ____ Days**	*Including 1^st^ day*
		starting ___/ ___/ ___	
		minimum TIME GAP _________ h	*Interval between fractions*
	**DOSE-RATE ______**	**Gy/min**	***Mean LUNG dose-rate:** Lung dose per fraction divided by lung irradiation time per fraction*

III	**Normal tissues**		***Other organs at risk***
		ORGAN DOSES	
		_________________: _________ Gy _________________: _________ Gy	*DD/D ≥ ± 10% of TBI-dose due to shieldings or additional beams*
	3. _________________: _________ Gy		

IV	**Serial TBI**	**Irregular parameters** Dosage-timing matrix	***Not all beams, modifyers** applied in each fraction*
		______________________________ ______________________________ ______________________________	*Parameters not the same, in all fractions, or mixed TBI-techniques*

V	Notes		

*Dose modification:* Fluence modifying techniques are used, e.g, flattening filters in wide angle collimator TBI or sweeping beam TBI, or a wedge filter for oblique incidence of the beam. The influences of irregular body contours have to be compensated: for a.-p.-p.-a.-TBI, water substituting bolus material is used in front of the neck to limit the larynx dose; and for bilateral TBI, to limit the dose to head, neck and legs. The skin is a part of the target volume. A low skin dose has to be boosted, e.g, by scatter electrons from a suitable material positioned close to the patient's body, e.g, a scatter screen (spoiler) for accelerator photons, or a (sterile) coverlet for ^60^Co-photons. Small parts of the thorax wall receiving a slightly lower dose generally do not require additional local irradiation (e.g, with electrons).

***Sparing the lungs:*** To *reduce the lung dose*, e.g, to 80% of the target dose, individually shaped shields of calculated thickness are used, attenuating the primary radiation fluence to 60-70%, with respect to lung density and scatter radiation from surrounding tissues. If transmission shieldings are directly fixed to the skin and a.-p.-p.-a.-TBI is applied in a supine/prone position, the patient can be positioned relaxed and no fixation or additional verification is needed. Suitable stacks are of lead rubber cutouts or of thin lead sheet cutouts. The *lung dose rate can be reduced* by a lead filter (for ^60^Co) or by decreasing the accelerators pulse repetition frequency.

***Localization:*** The measures of the patient's body have to be determined for each patient position (e.g, in prone and supine position or lying on the side). At least, the diameters of relevant parts of the body (in beam direction and across, e.g, through all dose points given in Figure 3 have to be determined. For the thorax region at least three pairs of tomograms are needed to evaluate mean values of lung thickness, lung density and thorax wall thickness. The shape of lung shieldings has to be documented and verified under TBI conditions.

***Dose calculations:*** For TBI treatment planning, it is usual to perform dose calculations for selected dose points [Figure 3]. To determine the absorbed dose, all physical influences have to be taken into account: the dose monitor calibration, the fluence profile (along the typical position of the body midline and across) and the local influences by parts of the body: mean thickness and the corresponding scatter volume. (The scatter dose contributions can be determined using the beam-zone method.[6] For lung dose determination, relevant influence parameters are lung thickness, mean lung density, thorax wall thickness, material and thickness of lung shields; lung volume (size and thickness), scatter volume (partial body size and thickness); and the volume of the shieldings.

## 5. TBI dosimetry

The absorbed dose to water is the measurand for the radiation dose; unit is Gray (Gy).

*All dosimetric measurements* have to be performed under physical and technical conditions used in TBI, especially the calibration of the TBI dose monitor and the calibration of dosemeters for absolute and relative TBI dosimetry, taking into account all influences of scatter radiation from floor or walls and the reduced scatter dose from the distant treatment head, so as influences by dose modifyers or technical modifications.

***Dosemeter:*** Detector probes and cables must be free of stem or cable effects. All devices for absolute dosimetry must be calibrated traceable to a national standard and for the radiation quality used in TBI. Detectors for concurrent *(in-vivo)* dosimetry during TBI (typically semiconductor detectors) have to be calibrated under the conditions of use. The correct function of the device has to be verified by the user prior to application.

*The TBI dose monitor should be calibrated under TBI conditions* against the dose at the center of a sufficiently large regular (e.g, square) water-substituting phantom: at least 30 × 30 cm in size and with a thickness of 20 cm to represent the typical situation of attenuation and scatter for a.-p. - p.-a.-TBI of adults. (The proposal of AAPM Report No. 17 to refer all dosimetric TBI data to those in a half infinite phantom cannot be recommended, as direct dose measurements under extrapolated conditions are not realistic.) *TBI dose monitor* is the accelerator dose monitor or the cobalt unit timer for fixed beam TBI and the inverse of the velocity in moving beam TBI. In patient translation TBI with a planned constant velocity,[6] the product of dose and velocity is a calibration constant; here the timer or accelerator dose monitor and the preset travel distance have to guarantee to irradiate the whole body length. All relative dose data have to be referred to calibration conditions.

***Systematic relative dosimetry - the beam parameters:*** Two dose profiles have to be measured, one longitudinally and one transverse to the body midline at the typical patient's midline distance, thus, the main axes of the beam or the diagonals, depending on the orientation of the patient. To measure these fluence profiles relative to the center of the beam, an ionisation chamber with a suitable small build-up cap simulating a tissue depth of 10 cm is scanned through the beam, free in air. For extreme inhomogeneous beam fluence profiles, an additional flattening filter may be needed. For TBI-techniques such as sweeping-beam TBI or TBI with adjacent multiple beams, the irradiation distance and the angle of beam incidence varying along the body have to be taken into account.

***Systematic relative dosimetry - the body parameters:*** The attenuation of the primary radiation and the contribution of scatter radiation - or missing scatter - can best be determined as a function of body thickness and effective width, by measuring the dose at the center of regular (square) water-substituting phantoms of different sizes (5^2^ cm^2^ - 50^2^ cm^2^) and thicknesses (10-30 cm, as typical values for a.-p.-p.-a.-TBI) and relative to the dose under calibration conditions. Dosimetric measurements within large automated water phantoms do not represent the final body size. Depth dose distributions are of less interest in a.-p. - p.-a.-TBI, as the opposing beam depth dose distributions are almost flat. The skin dose has to be determined at the entrance and exit side, taking into account the influences of all beams and dose-modifying materials as well as backscatter from wall or floor.

***Lung dose:*** To determine the dose to the lungs, several influences have to be taken into account, e.g, by varying the parameters of a regular heterogeneous phantom: lung thickness (5-15 cm), mean lung density (0.1-0.5 g/cm^3^), thickness of thorax wall (1-8 cm), material and thickness of lung shields (e.g, corresponding to 10-50% dose reduction), size of the lung (5^2^ cm^2^-15^2^ cm^2^), size of the effective scatter volume of body parts (20^2^ cm^2^-50^2^ cm^2^) and shielding size (5^2^ cm^2^ - 15^2^ cm^2^).[6,33]

***3D-planning:*** At the moment, no commercial planning system is prepared for 3D-TBI-planning. Thus for planning systems, it has to be checked if the dose data measured at the isocenter and the algorithms applied are suitable for the special physical conditions in TBI. It also has to be verified that the dose within tissue heterogeneities or below shieldings, at the skin or at tissue interfaces, are calculated sufficiently precise.

### 6. Quality management

Dosimetric confirmation measurements in suitable heterogeneous body-shaped phantoms are needed prior to establishing new or modified TBI techniques or planning algorithms.

All prescribed and planned dose parameters should be verified and controlled independently by electronic means prior to and recorded during and after, each TBI treatment. The exact position of lung shieldings should be verified and documented prior to each fraction.

*In-vivo* dosimetry should be used while introducing TBI. Regular *in-vivo* dosimetry is needed if relevant irradiation treatment parameters can not be verified, controlled and recorded electronically or if parameters could change. As a consequence and to avoid the associated uncertainties, TBI techniques should be chosen not requiring regular *in-vivo* dosimetry.
